# Endovascular Treatment and Angiographic Characteristics of Aneurysms at the Origin of the Anterior Choroidal Artery

**DOI:** 10.3389/fneur.2022.832604

**Published:** 2022-03-14

**Authors:** Yiheng Wang, Jinlu Yu

**Affiliations:** Department of Neurosurgery, The First Hospital of Jilin University, Changchun, China

**Keywords:** anterior choroidal artery, aneurysm, angiography, anatomy, endovascular treatment

## Abstract

**Background:**

The information available about the variations and anatomy of the anterior choroidal artery (AchA) with aneurysm at the origin of the vessel and the outcomes of endovascular treatment (EVT) for AchA aneurysms is incomplete.

**Materials and Methods:**

A retrospective study of 54 consecutive patients who were admitted to our hospital with a diagnosis of AchA aneurysm and treated with EVT was performed. The variations and anatomy of the AchA and the outcomes of EVT for AchA aneurysms were analyzed.

**Result:**

The 54 patients were aged 35–82 years (mean age, 56.1 ± 19.7 years) and included 32 females (59.3%, 32/54). Regarding AchA anatomy, 63.5% of AchAs had a typical S-shaped course. The diameter of the AchA origin averaged 0.8 ± 0.3 mm. Of all the AchA aneurysms, 51.9% were ruptured. The diameter of AchA aneurysms averaged 4.1 ± 2.4 mm. Moreover, 40.7% of 54 cases had multiple aneurysms. EVT was assisted with stenting for 25.9% of 54 AchA aneurysms. An immediate Modified Raymond-Roy Classification grade of I was obtained in 96.3% of AchA aneurysm cases. After EVT, the ischemic complication rate was 13%. In total, 83% of patients had good outcomes, with a Glasgow Outcome Scale score of 4–5. Follow-up angiography showed acceptable treatment results in this study.

**Conclusion:**

The study showed that the AchA had a complex angiographic anatomy in cases with aneurysms at the origin of the vessel and that the anatomical features can be helpful in EVT. EVT for aneurysms at the origin of the AchA had good outcomes.

## Introduction

The anterior choroidal artery (AchA) is the branch arising from the internal carotid artery (ICA) after the origin of the posterior communicating artery (PcomA); it is a very important small vessel and has a complex anatomy (1, 2). The AchA supplies the optic tract, the lateral geniculate nucleus, the posterior two-thirds of the posterior limbs of the internal capsule, and the choroid plexus; the collateral circulation of this artery is poor (3). The classic features of AchA occlusion are known as AchA symptoms and include contralateral hemiplegia, hemianesthesia and hemianopsia (4). Therefore, a thorough understanding of AchA anatomy is very important.

Currently, the available data on AchA anatomy are mainly derived from studies of human anatomical specimens, intraoperative AchA observations or angiographic examinations in normal populations (4–7). The angiographic anatomy of the AchA has been elucidated in normal populations to a certain extent (5). However, the available information about the variations and anatomy of the AchA with aneurysm is incomplete. Therefore, this study examined the anatomy of the AchA in cases with an aneurysm at the origin of this vessel.

For aneurysms at the origin of the AchA (the junction between the ICA and AchA), endovascular treatment (EVT) is effective in preventing bleeding (8). However, reports focused on EVT of AchA aneurysms are limited. Therefore, this study included 54 patients with an aneurysm at the origin of the AchA who underwent EVT. At the same time, an anatomical study of the AchA was also performed in this pathological state.

## Materials and Methods

### Patient Information

A total of 54 patients in the Department of Neurosurgery of the First Hospital of Jilin University from September 2019 to March 2021 were continuously collected and retrospectively analyzed. All patients had AchA aneurysms and underwent routing EVT by coiling with or without stenting assistance. Flow divertor stenting for AchA aneurysms was excluded because the technique was different from routing EVT (9). The present study was approved by the Ethics Committee of our institute. The preoperative state was evaluated in cases of subarachnoid hemorrhage (SAH) with Hunt-Hess grade (10).

### Angiographic Characteristics of the AchA

**Figure 1:**
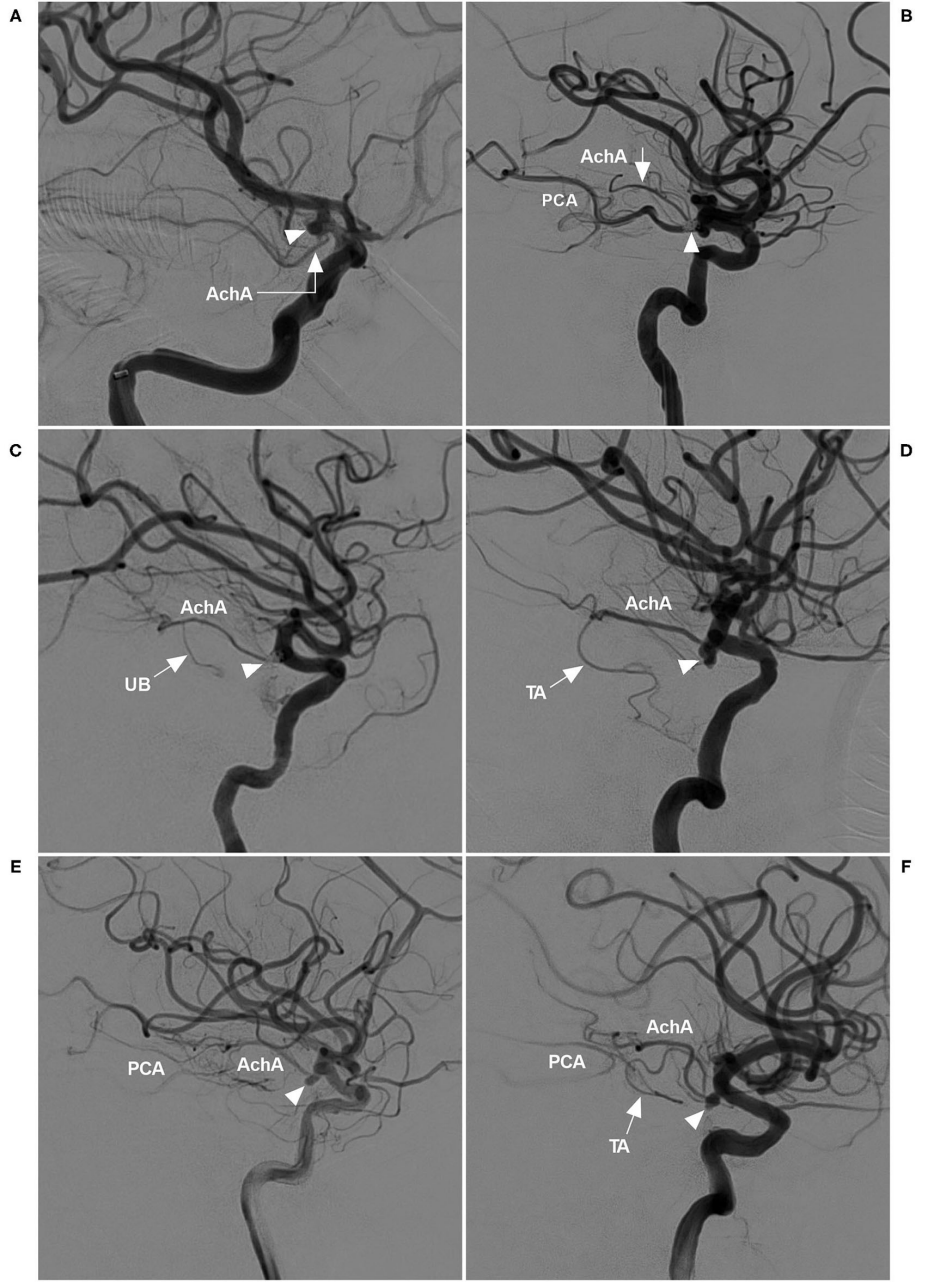
AchA anatomy in cases with aneurysm. **(A)** DSA showing a single-trunk AchA (right-angled arrow) with an aneurysm (triangle) at its origin. **(B)** DSA showing the AchA consisting of a plexus of vessels with a coiled aneurysm (triangle) at its origin. In this plexus, a major branch (arrow) was prominent; moreover, PCA was the fetal type. **(C)** DSA showing a large AchA with a coiled aneurysm (triangle), in which an uncal branch (UB, arrow) could be seen. **(D)** DSA showing a large AchA with an aneurysm (triangle), in which a temporal artery (TA, arrow) could be seen. **(E)** DSA showing a large AchA with an aneurysm (triangle). The AchA anastomoses with the PCA. **(F)** DSA showing a large AchA with an aneurysm (triangle). The AchA sent out a large temporal artery (TA, arrow) and anastomosed with the PCA. AchA, anterior choroidal artery; DSA, digital subtraction angiography; PCA, posterior cerebral artery; TA, temporal artery; UB, uncal branch.

The AchA can be a single artery, duplicate arteries, or a plexus of vessels; it can also be a large single artery that sends out a cortical branch, which divides into the uncal and temporal branches (1, 5). The temporal branch can anastomose with the posterior cerebral artery. These classifications were recorded (Figure 1).

**Figure 2:**
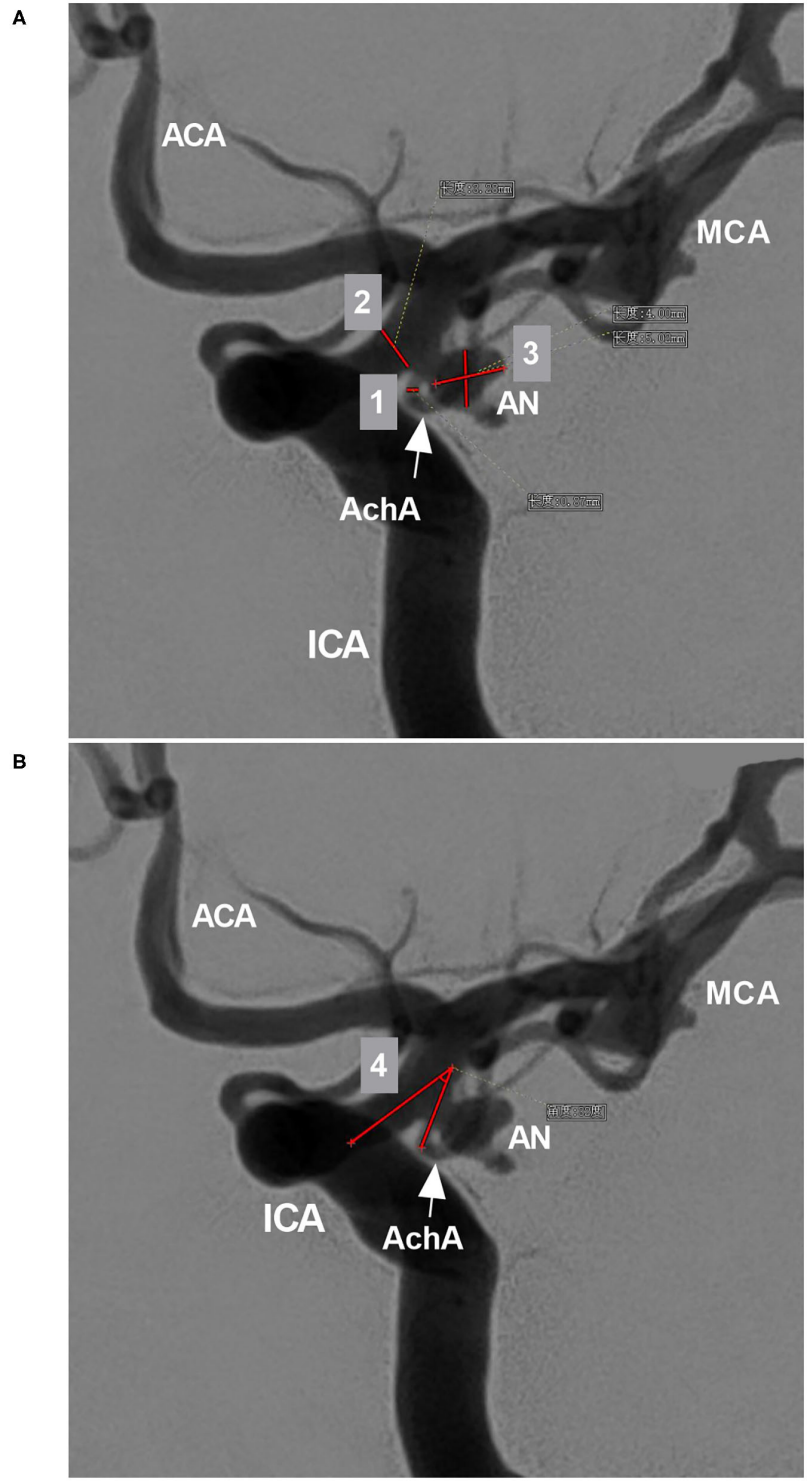
Parameter measurement. **(A)** DSA showing the measurement diameters: number 1 refers to the diameter of the AchA at the origin (arrow), number 2 refers to the diameter of the ICA at the AchA origin, and number 3 shows the measurement of the width and length of the aneurysm (AN). **(B)** DSA showing the degree (number 4) between the ICA and AchA at the origin (arrow). ACA, anterior cerebral artery; AchA, anterior choroidal artery; AN, aneurysm; DSA, digital subtraction angiography; ICA, internal carotid artery; MCA, middle cerebral artery.

The AchA often follows an S-shaped course and consists of cisternal and plexal segments. The plexal segment can present with a single branch, two branches or a plexus (5). In this study, the AchA course and plexus segment branching were recorded. The diameters of the AchA origin and the ICA at the AchA origin were measured (Figure 2A), and the angle between the ICA and AchA origin was measured (Figure 2B).

### Characteristics of AchA Aneurysms

**Figure 3:**
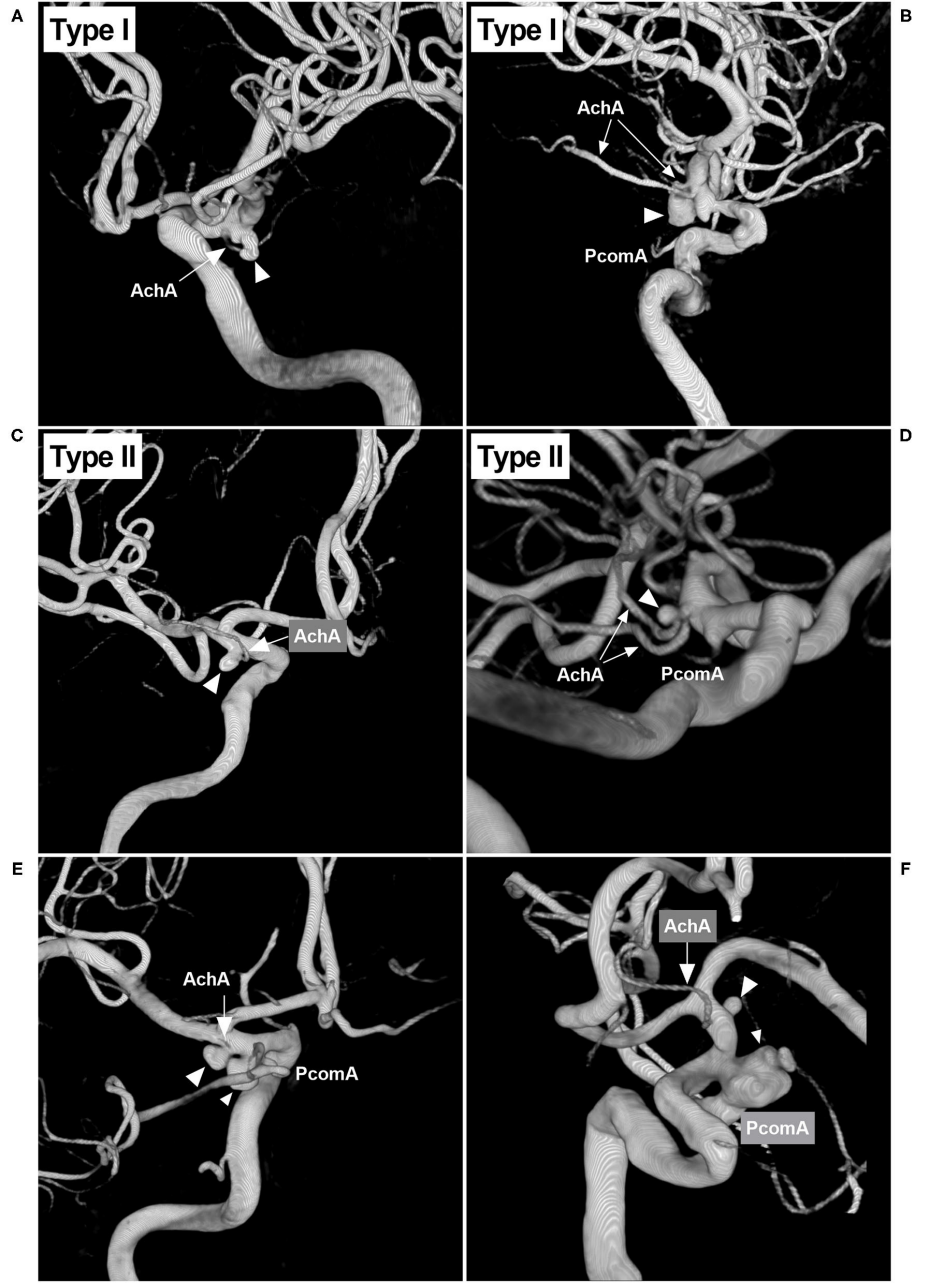
Classifications of the AchA aneurysm and associated aneurysms. **(A)** 3D-DSA showing an AchA aneurysm (triangle): a single-trunk AchA (arrow) was located away from the aneurysm neck; this type of aneurysm belongs to type I. **(B)** 3D-DSA showing an AchA aneurysm (triangle): a duplicate AchA (arrows) was located away from the aneurysm neck. This type of aneurysm belongs to type I, and a PcomA can be seen. **(C)** 3D-DSA showing an AchA aneurysm (triangle): a single-trunk AchA (arrow) arose from the aneurysm neck; this type of aneurysm belongs to type II. **(D)** 3D-DSA showing an AchA aneurysm (triangle): a duplicate equal AchA (arrows) arose from the aneurysm neck; this type of aneurysm belongs to type II, and a PcomA can be seen. **(E)** 3D-DSA showing the AchA aneurysm (large triangle) and a neighboring PcomA aneurysm (small triangle); they are close, and the arrow indicates the AchA. **(F)** 3D-DSA showing the AchA aneurysm (large triangle) and a neighboring large PcomA aneurysm (triangle); they are far, the arrow indicates the AchA. AchA, anterior choroidal artery; DSA, digital subtraction angiography; PcomA, posterior communicating artery; 3D, three-dimensional.

The width and length of the aneurysms were measured, and then the mean diameter was calculated (length plus width divided by two) (Figure 2A). The aneurysms were divided into two types: when the AchA arose from the ICA and was distanced from the aneurysm neck, the aneurysm belonged to type I (Figures 3A,B); when the AchA arose from the aneurysm neck, the aneurysm belonged to type II (Figures 3C,D). In addition, the associated aneurysms were also recorded (Figures 3E,F).

In hemorrhagic patients, the AchA aneurysm was classified as ruptured according to the following criteria: only the AchA aneurysm was found, SAH focused on AchA aneurysm, or when the AchA aneurysm had adjacent aneurysms, AchA aneurysm had an irregular shape or daughter sac (11). Otherwise, the AchA aneurysm was considered unruptured.

### Scheme of EVT

#### Medication Management

For narrow-necked aneurysm, single coiling was planned, and antiplatelet medication was unnecessary. For wide-necked aneurysm, when stent assistance was planned, patients with unruptured aneurysms were given dual-antiplatelet medication with aspirin 100 mg and clopidogrel 75 mg for a minimum of 3–5 days before EVT. For patients with ruptured aneurysms, a loading dose of dual-antiplatelet medication, consisting of aspirin 300 mg and clopidogrel 300 mg, was given for a minimum of 3 h before EVT. After EVT, dual-antiplatelet therapy was maintained for 1–3 months. Then, 100 mg aspirin was given for a minimum of 6 months or for life.

#### EVT Procedure

All patients were treated under general anesthesia via a transfemoral approach. First, digital subtraction angiography (DSA) was used to assess the angioarchitecture of the aneurysm and the AchA. Then, under roadmap guidance, a 0.014-inch microcatheter that delivered coils with appropriately shaped tips was placed into the aneurysm to perform coiling. For wide-necked aneurysms, the microcatheter that delivered the stent was passed through the aneurysm neck and stably maintained in the straight segment of the middle cerebral artery (MCA) to release the stent. During EVT, the AchA must be preserved to avoid a disastrous consequence.

### Evaluation of Outcomes

The complications and resolutions, as well as the length of hospital stay, were recorded. The Glasgow Outcome Scale (GOS) was used to evaluate the outcome at discharge (12). Immediate and follow-up DSA was performed to examine the outcome of aneurysm coiling with the Modified Raymond-Roy Classification (MRRC), as follows: Class I: complete obliteration; Class II: residual neck; Class IIIa: residual aneurysm with contrast within coil interstices; and Class IIIb: residual aneurysm with contrast along the aneurysm wall (13).

**Figure 4:**
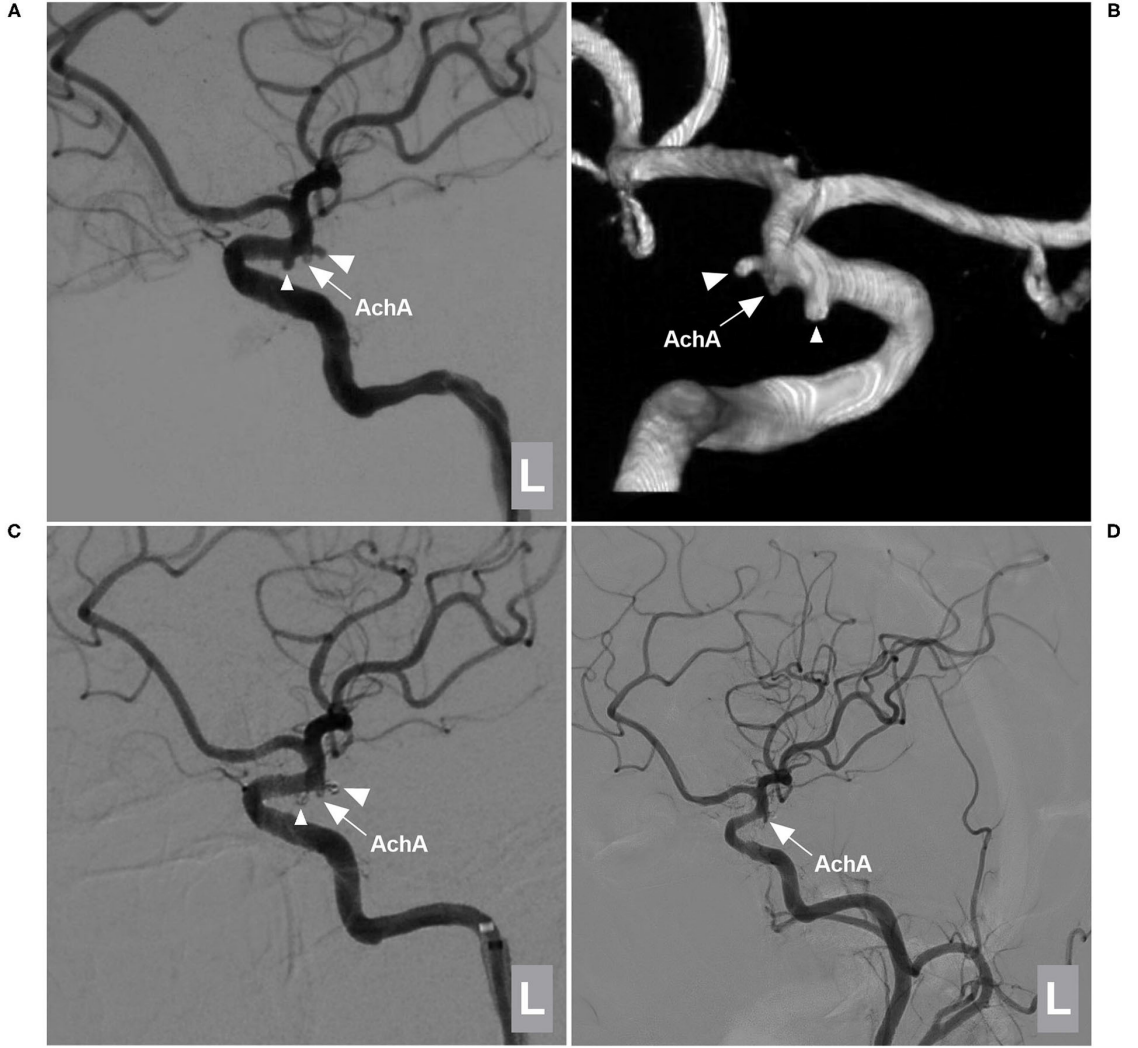
EVT of a type I AchA aneurysm associated with PcomA aneurysm. **(A,B)** DSA **(A)** and 3D-DSA **(B)** of the left ICA showing the AchA (large triangle) and PcomA (small triangle) aneurysms; the arrow indicates the AchA origin is away from the aneurysm neck. **(C)** DSA of the left ICA showing that the two aneurysms (large and small triangles) were coiled under stent assistance and that the AchA (arrow) was intact. **(D)** Follow-up DSA showing no recurrence of these two aneurysms with MRCC grade I, the AchA (arrow) was shown. AchA, anterior choroidal artery; DSA, digital subtraction angiography; EVT, endovascular treatment; ICA, internal carotid artery; L, left; MRCC, Modified Raymond-Roy Classification, PcomA, posterior communicating artery, 3D, three-dimensional.

**Figure 5:**
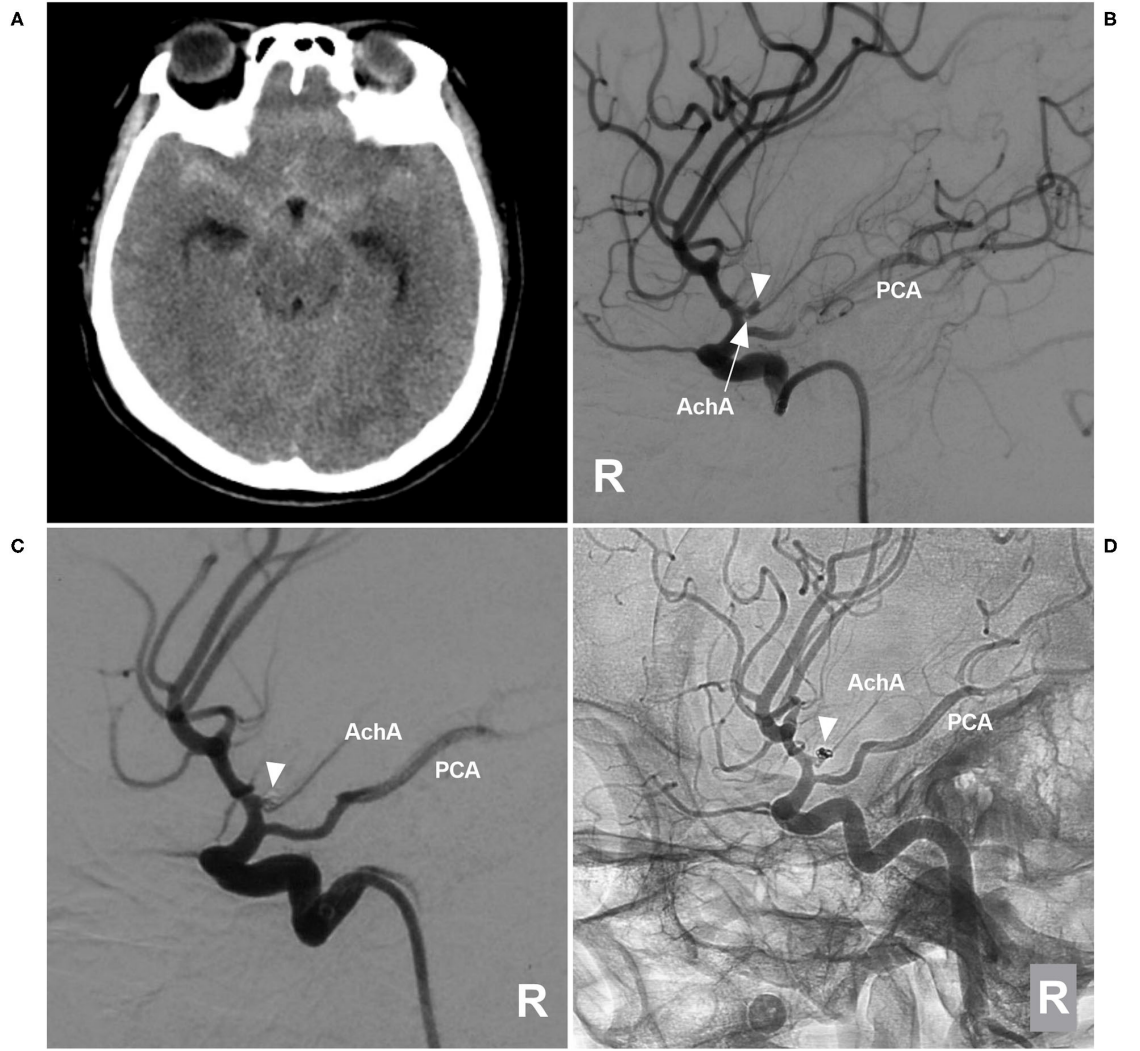
EVT of a type II AchA aneurysm. **(A)** CT showing subarachnoid hemorrhage. **(B)** DSA of the right ICA showing a type II AchA aneurysm (triangle); the AchA (arrow) arose from the aneurysm neck, and the PCA was the fetal type. **(C)** DSA of the right ICA showing that the aneurysm (triangle) was coiled, with MRCC grade I, the AchA was unblocked. **(D)** Follow-up unsubtracted DSA of the right ICA showing that the aneurysm (triangle) had no recurrence. AchA, anterior choroidal artery; CT, computed tomography; DSA, digital subtraction angiography; EVT, endovascular treatment; ICA, internal carotid artery; MRCC, Modified Raymond-Roy Classification; PCA, posterior cerebral artery; R, right; 3D, three-dimensional.

**Figure 6:**
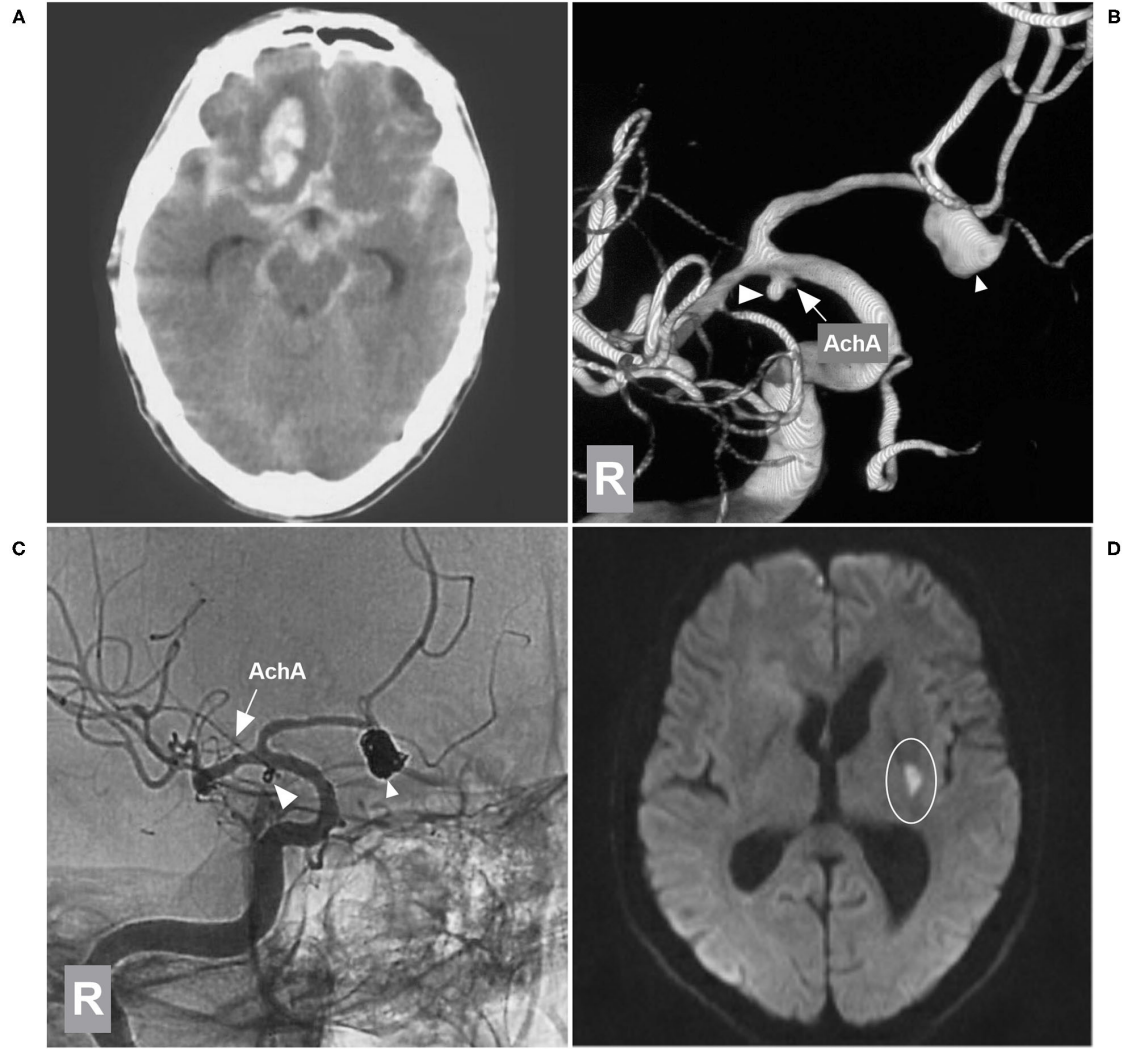
EVT of the AchA aneurysm with brain infarction. **(A)** CT showing subarachnoid hemorrhage with a hematoma in the right frontal lobe base. **(B)** 3D-DSA of the right ICA showing type II AchA aneurysm (large triangle) and AcomA aneurysm (small triangle), and the arrow indicates the AchA. **(C)** Unsubtracted DSA of the right ICA showing that two aneurysms were coiled (large and small triangles); the AchA (arrow) was intact. **(D)** MRI DWI sequence showing the acute infarction (circle region) in the posterior limb of the internal capsule. AchA, anterior choroidal artery; AcomA, anterior communicating artery; CT, computed tomography; DSA, digital subtraction angiography; DWI, diffusion-weighted imaging; EVT, endovascular treatment; ICA, internal carotid artery; MRI, magnetic resonance imaging; R, right; 3D, three-dimensional.

**Figure 7:**
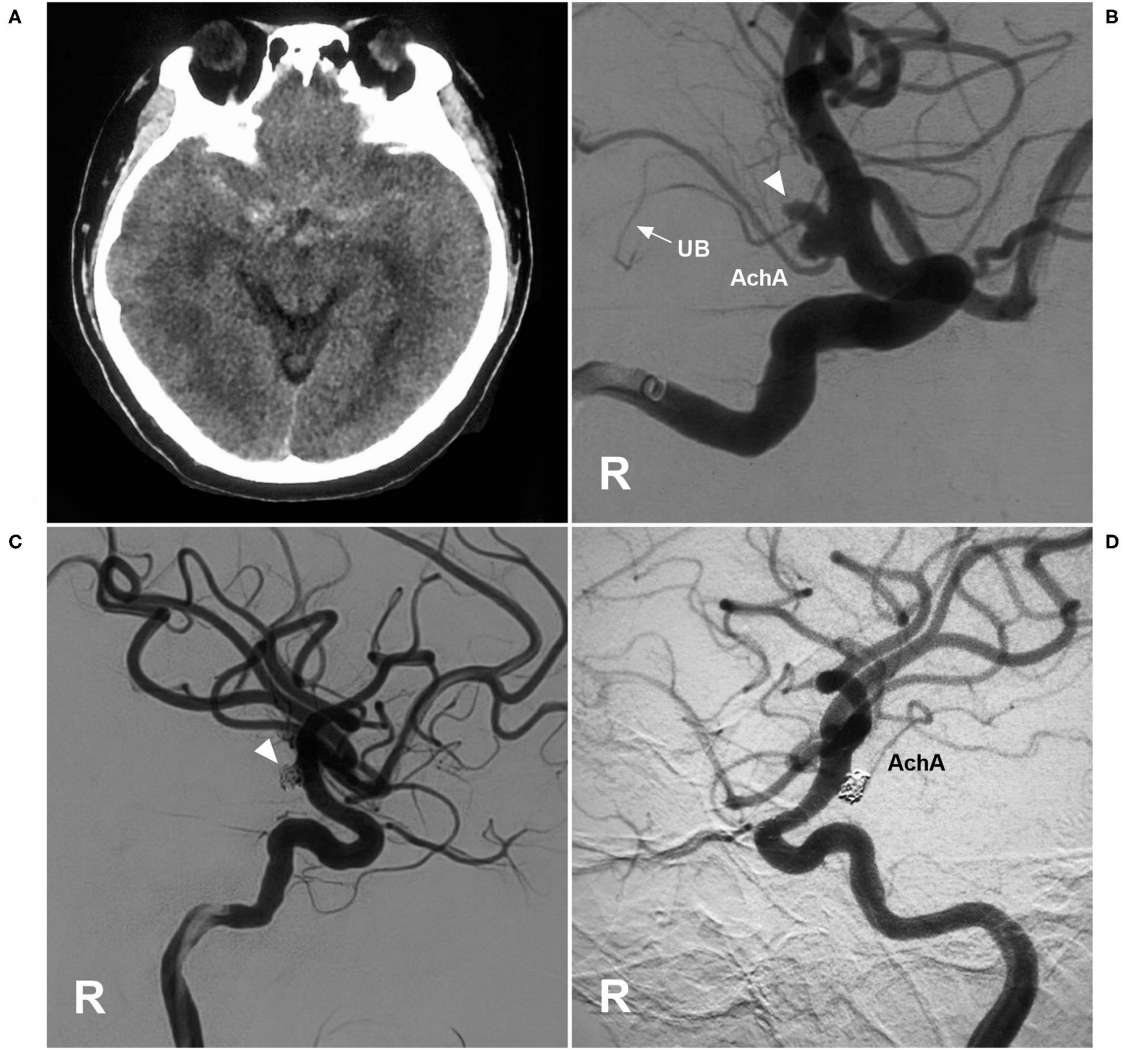
EVT of an AchA aneurysm with AchA occlusion. **(A)** CT showing subarachnoid hemorrhage. **(B)** DSA of the right ICA showing a type II AchA aneurysm (triangle). The AchA was hyperplastic, and the uncal branch (UB, arrow) could be seen. **(C)** DSA of the right ICA showing that the aneurysm was coiled (triangle); however, the AchA was occluded, without new neurological deficits. **(D)** Follow-up DSA showing the aneurysm with embolization with MRCC grade I, and the AchA reappeared. AchA, anterior choroidal artery; CT, computed tomography; DSA, digital subtraction angiography; EVT, endovascular treatment; ICA, internal carotid artery; MRCC, Modified Raymond-Roy Classification; R, right; UB, uncal branch.

**Figure 8:**
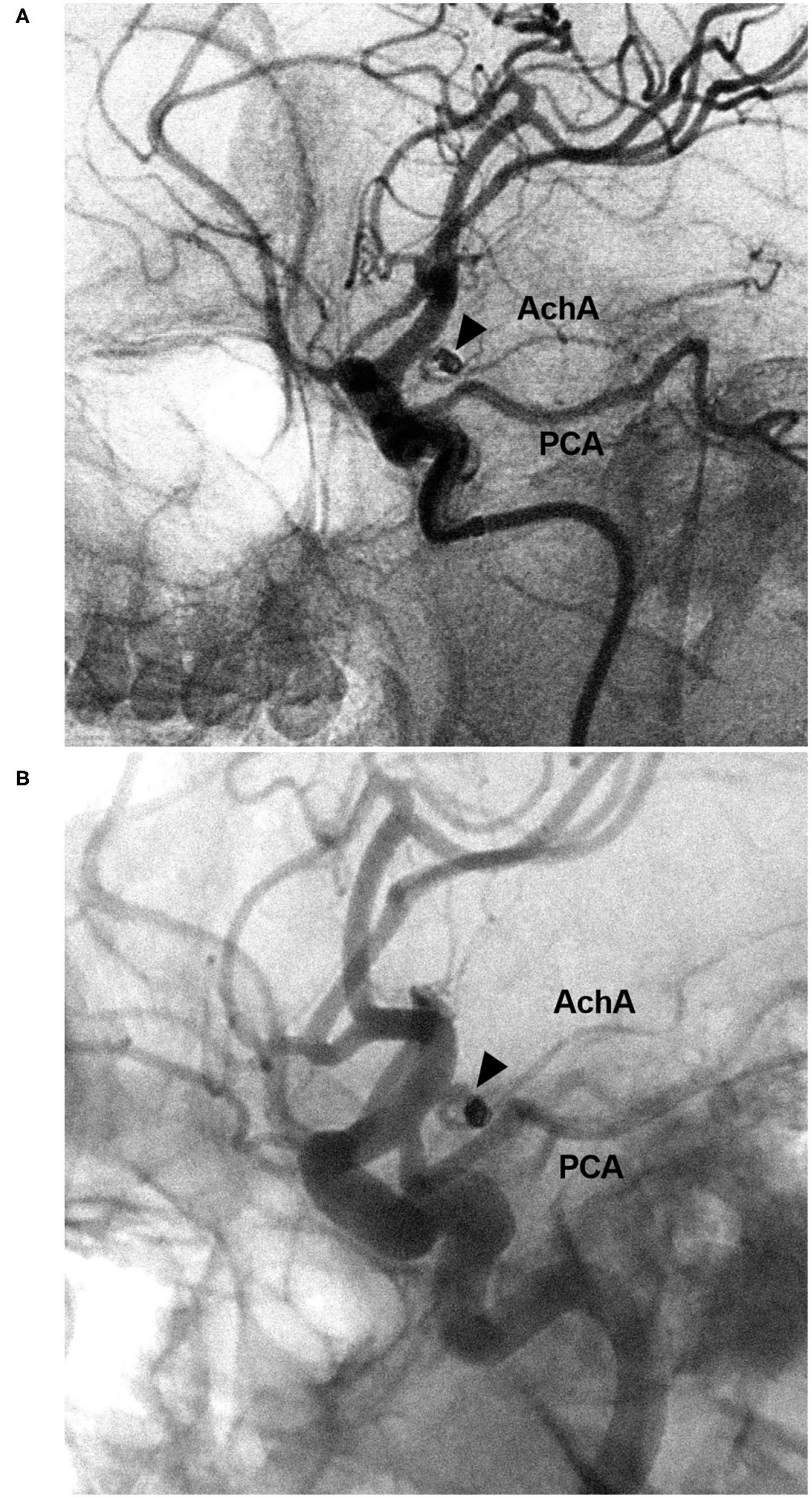
EVT of the AchA aneurysm with recurrence. **(A)** DSA of the ICA showing a type II AchA aneurysm (triangle) that was coiled with MRCC grade I. **(B)** Follow-up DSA showing aneurysm recurrence (triangle), the coils were compressed, with MRCC grade II. AchA, anterior choroidal artery; DSA, digital subtraction angiography; EVT, endovascular treatment; ICA, internal carotid artery; MRCC, Modified Raymond-Roy Classification; PCA, posterior cerebral artery.

In this study, some typical and educational cases of the EVT were provide (Figures 4–8).

## Results

### General Patient Data

Among the 54 cases, 16 (29.6%, 16/54) had no bleeding: six cases of occasional findings, four cases of headaches and six cases of dizziness. Moreover, 38 (70.4%, 38/54) cases had bleeding: 36 cases of SAH, 1 case of SAH + intracerebral hematoma and 1 case of SAH + intraventricular hemorrhage. Among 38 cases of bleeding, five were Hunt-Hess grade I, 25 were grade II, seven were grade III and 1 was grade IV.

### Angiographic Characteristics of the AchA

Of the 54 AchAs, 3 (5.6%, 3/54) AchAs had a duplicate branch (with equally developed branches in 1; with one stronger branch in 2). After two AchAs with equal duplicate branching were excluded, 33 (63.5%, 33/52) of the remaining 52 AchAs were found to have a typical S-shaped course of the cistern segment. Furthermore, in the 52 AchAs, the diameter of the AchA origin was 0.3 mm to 1.6 mm (mean, 0.8 ± 0.3 mm), and the angle between the ICA and AchA origin was 0–157 degrees (mean, 51.3 ± 39.5 degrees).

### Characteristics of the AchA Aneurysms

Of 54 AchA aneurysms, 34 (63%, 34/54) aneurysms were located on the left side, 28 (51.9%, 28/54) aneurysms were ruptured. There were 5 (9.3%, 5/54) type I AchA aneurysms and 49 (90.7%, 49/54) type II AchA aneurysms.

In 54 cases with AchA aneurysms, 22 cases (40.7%, 22/54) were associated with multiple aneurysms (total 24 aneurysms) as follows: 11 ipsilateral PcomA aneurysms, four ipsilateral MCA aneurysms, three ipsilateral ophthalmic artery aneurysms, three distal anterior communicating artery aneurysms, one ipsilateral A1 anterior cerebral artery aneurysm, one contralateral PcomA aneurysm, and one contralateral MCA aneurysm.

Of the 54 patients with AchA aneurysms, five had moyamoya disease (9.3%, 5/54), two had contralateral ICA occlusion (3.7%, 2/54), one had primitive trigeminal artery (1.9%, 1/54), and one had accessory MCA (1.9%, 1/54). Among the 54 ICA sides with AchA aneurysms, 31 PcomAs (57.4%, 31/54) were found, of which 15 were hyperplastic.

### Results of EVT

EVT was assisted with stenting for 14 of the 54 AchA aneurysms (25.9%, 14/54). During EVT, one AchA was found to be occluded, and tirofiban was given; however, no angiographic improvement was obtained, and no new deficit was observed after EVT (Figure 7). When EVT was finished, an immediate MRCC grade I was obtained for 52 AchA aneurysms (96.3%, 52/54), and grade II was obtained for two aneurysms (3.7%, 2/54).

At the same time, in 12 cases with 24 associated aneurysms, 12 (50%, 12/24) aneurysms underwent EVT, and the others received follow-up or had to wait for subsequent treatment. After EVT, there were seven ischemic complications (13.0%, 7/54), which were all Type II aneurysms with the single trunk AchA type. Then, treatments including hypertension, hypervolemia and hemodilution were given.

The length of hospital stay ranged from 3 to 22 days (16.3 ± 4.7 days). Upon discharge, 21 patients (38.9%, 21/54) had GOS scores of 5, 24 patients (44.4%, 24/54) had GOS scores of 4, eight patients (14.8%, 8/54) had GOS scores of 3, and one patient (1.9%, 1/54) had GOS scores of 2. Among the 54 cases, 15 cases (27.8%, 15/54) had follow-up angiography from 3 to 6 months postoperative. One AchA aneurysm with MRCC grade I recurred with an increase in grade to MRCC grade II (Figure 8). Two AchA aneurysms with MRCC grade II remained stable. In the case shown in Figure 7, the occlusion reappeared. The detail clinical data are shown in Table 1.

**Table 1:** Main clinical data of the patients in the present study.

**Age (years)**
Mean	56.1 ± 19.7
Range	35–82
**Female/male**	1.6 (32/22)
**Presentation**
**Hemorrhagic**	**29.6% (16/54)**
Non-hemorrhagic	70.4% (38/54)
**Angiographic characteristics of the AchA**	
**Cisternal segment**	
A single trunk	42.6% (23/54)
A plexus with a major branch	16.7% (9/54)
Duplicate branches	5.6% (3/54)
Uncal branch	31.5% (17/54)
Temporal branch	3.7% (2/54)
Typical S-shaped course	63.5% (33/52)
Atypical S-shaped course	36.5% (19/52)
**Plexal segment**	
Main single branch	53.7% (29/54)
Two branches	25.9% (14/54)
A plexus	20.4% (11/54)
**Diameter of the AchA origin (mm)**	
Mean	0.8 ± 0.3
Range	0.3–1.6
**Diameter of the ICA at the AchA origin (mm)**	
Mean	4.5 ± 0.8
Range	2.3–6.3
**Angle between the ICA and AchA origin (degree)**	
Mean	51.3 ± 39.5
Range	0–157
**Characteristics of the AchA aneurysm**	
Ruptured/unruptured	1.1 (28/26)
**Diameter of AchA aneurysm (mm)**	
Mean	4.1 ± 2.4
Range	1.5–13
Type I/II	1.0 (5/49)
Multiple aneurysm	40.7% (22/54)
**Results of EVT**	
Stent-assistance	25.9% (14/54)
**Immediate MRCC**	
Grade I	96.3% (52/54)
Grade II	3.7% (2/54)
**Ischemic complications**	13.0% (7/54)
**Glasgow outcome scale**	
5	38.9% (21/54)
4	44.4% (24/54)
3	14.8% (8/54)
2	1.9% (1/54)

*AchA, anterior choroidal artery; EVT, endovascular treatment; ICA, internal carotid artery; MRCC, Modified Raymond-Roy Classification*.

## Discussion

The AchA is very small compared to its length and the areas it supplies. An anatomical study in a normal population was reported (1, 4, 14). However, the available information about the variations in and anatomy of the AchA associated with aneurysms at the junction of the ICA and AchA is incomplete (15). The prerequisite for angiographic anatomy in this study was an AchA with an aneurysm to try to address this issue.

Could an aneurysm in the AchA origin affect the morphology of the AchA? In this study, the typical S-shaped course occurred in 63.5% of cases, the uncal branch occurred in 31.5%, and a single main trunk of the plexal segment occurred in 55.8%, compared with 71.8, 35.7, and 62% in a large-scale angiographic study of normal angiographic anatomy of the AchA by Takahashi et al.; these results were all similar, which indicated that the associated aneurysm may not change the course and branching of the AchA (5).

Could an aneurysm in the AchA origin affect the diameter of the AchA? In this study, the diameter averaged 0.8 mm (range 0.3–1.6 mm), compared with 0.5–1.5 mm in a study of specimens by Rhoton et al. (14) and 0.75 mm (range, 0.28–2.0 mm) in an angiographic study by Takahashi et al. (5); these results were all similar, which indicated that the associated aneurysm may not change the diameter of the AchA origin.

In this study, some of the data were focused on the aneurysms themselves. AchA aneurysm is rare, representing 2–5% of all intracranial aneurysms (16, 17). Some AchA aneurysms can be associated with other aneurysms (18). In our study, the rate was 40.7% (22/54), which made the treatment complex. Currently, EVT is comparable or superior to surgical clipping for the treatment of most intracranial aneurysms including AchA aneurysms (16, 19). However, EVT of AchA aneurysms can cause an ischemic complication, the main cause was occlusion or ischemia of the AchA (6). Therefore, the classifications of the AchA aneurysms are very important.

In a study by Aoki et al., the classification of AchA aneurysms was as follows: type A (artery type), AchA arose directly from the ICA; type B (neck type), AchA arose from the aneurysmal neck; type C (dome type), AchA arose from the aneurysmal dome; and type D (truncal type), the aneurysm originate in a part of the AchA itself (16). For EVT application, the classification can be simplified, and Types B-D can be merged into one type. Similar to our study, type A was type I in our study, type B-D AchA aneurysms belonged to type II.

Apparently, EVT is safe for type A or I aneurysms, because the AchA is not near the aneurysm neck (20). However, there were fewer type A or I aneurysms. In our study, the rate was 9.3%. In the report by Aoki et al., type A aneurysms accounted for 15.6% (16). Therefore, most AchA aneurysms belonged to type II. In our study, type II AchA aneurysms are also appropriate for the EVT, because all AchAs arose from the aneurysm neck, most EVT can preserve the AchA. During EVT, even when the AchA is intact, ischemic complications can still occur (21). In our study, ischemic complications occurred at a rate of 13%, with all AchA preservation in type II, and the reason may be disturbance of AchA blood flow from the EVT.

The AchA may arise from the different orientations of the ICA. For instance, in a study by Gibo et al., the AchA arose from the ICA, with 66% arising from the posterolateral aspect, 28% from the posteromedial aspect and 6% from the lateral aspect (22). In this study, the angle between the ICA and AchA origin was 0–157 degrees (mean 51.3 ± 39.5), which indicated that the AchA origin varied from the ICA. When the AchA originated from the inferior corner between the ICA and aneurysm, the angle was the smallest, and the angle was the largest when the AchA originated from the superior corner between the ICA and aneurysm. Due to an inappropriate degree of projection in EVT, especially for an AchA arising from the aneurysm flank, the AchA often cannot be seen clearly, which adds to the risk of AchA occlusion.

The AchA has been considered to be one the most dangerous arteries for EVT because of the risk of serious ischemic complications (17). The occurrence of ischemic complications depends on AchA collateral circulation (23). In this study, 35.2% of AchAs had cortical branches, indicating collateral compensation due to good development. In the case of Figure 7, the AchA had an uncal branch; after the AchA was occluded, no complications occurred, which was associated with good collateral circulation. In theory, the type of AchA can affect the EVT outcome. When AchA presents with a single trunk, the risk is higher; for instance, our cases with ischemic complications are all the single-trunk type of AchA.

To preserve the AchA to prevent ischemic complications, after the saccular aneurysm body was densely coiled, the neck remnant can be allowed to remain as an alternative to complete aneurysmal occlusion with AchA occlusion (8). In this study, 3.7% of cases had an immediate MRCC grade of II to avoid the AchA occlusion. In our study, the routing EVT of the coiling with/without low-metal-coverage stenting assistance brought a satisfactory result, and the prognosis was acceptable (83.3% of patients had a GOS of 4–5).

For the EVT for AchA aneurysms, although the AchA was intact and unblocked, the AchA ischemia can occur due to the perfusion insufficiency. So, intraoperative monitoring was necessary (16). Transcranial motor-evoked potential (MEP) monitoring is useful for detecting blood flow insufficiency in the AchA and reflects motor function during coil embolization of AChA aneurysms, because AChA blood flow detected by angiography does not always reflect MEP status (24). Decreases in MEP amplitude during EVT for AChA aneurysms are likely to reflect motor dysfunction (25).

In this study, we did not favor flow-diverting stents for AchA aneurysms. On the one hand, in our study, many aneurysms (46.1%, 26/54) were ruptured, not too large (mean diameter 4.1 mm), or inappropriate for a flow-diverting stent; on the other hand, flow-diverting stents may result in AchA occlusion (26, 27). The diameter of the ICA at the AchA averaged 4.5 ± 0.8 mm, which was appropriate for conventional intracranial stents (28).

In summary, our study showed that the AchA had a complex angiographic anatomy in cases with AchA origin aneurysms and that the anatomical features can be helpful in EVT. EVT for aneurysms of AchA origin had a good outcome.

## Limitation

This is a retrospective study with a limited sample size, and the conclusion in this study should be interpreted cautiously. The rate of angiographic follow-up in this study was low, which makes it difficult to evaluate the long-term efficacy of EVT.

## Data Availability Statement

The raw data supporting the conclusions of this article will be made available by the authors, without undue reservation.

## Ethics Statement

The studies involving human participants were reviewed and approved by the Ethics Committee of the First Hospital of Jilin University. The patients/participants provided their written informed consent to participate in this study. Written informed consent was obtained from the individual(s) for the publication of any potentially identifiable images or data included in this article.

## Author Contributions

JY contributed to the conception and design of the manuscript and critically revised the manuscript. JY and YW wrote the manuscript and collected the medical records of the patients. Both authors approved the final version of this manuscript.

## Conflict of Interest

The authors declare that the research was conducted in the absence of any commercial or financial relationships that could be construed as a potential conflict of interest.

## Publisher's Note

All claims expressed in this article are solely those of the authors and do not necessarily represent those of their affiliated organizations, or those of the publisher, the editors and the reviewers. Any product that may be evaluated in this article, or claim that may be made by its manufacturer, is not guaranteed or endorsed by the publisher.
